# Overexpression of HPIP as a biomarker for metastasis and prognosis prediction in endometrial cancer patients

**DOI:** 10.1002/jcla.22959

**Published:** 2019-06-26

**Authors:** Li Cheng, Tingting Zhao, Shiguo Li, Yao Wang, Hui Fei, Fanling Meng

**Affiliations:** ^1^ Department of Obstetrics and Gynecology The Seventh Affiliated Hospital, Sun‐Yat sen University Shenzhen Guangdong Province China; ^2^ Department of Obstetrics and Gynecology The Daqing Oilfield General Hospital Daqing Heilongjiang China; ^3^ Department of Gynaecology Harbin Medical University Cancer Hospital Harbin Heilongjiang China

**Keywords:** endometrial cancer, hematopoietic pre‐B‐cell leukemia transcription factor, HPIP, metastasis, prognosis

## Abstract

**Background:**

Hematopoietic pre‐B‐cell leukemia transcription factor (PBX)‐interacting protein (HPIP) has shown to be overexpressed in several human cancers. The purpose of this study was to explore the expression of HPIP in endometrial cancer (EC) and its associated effects on disease.

**Methods:**

A total of 113 EC patients at the Harbin Medical University Cancer Hospital between August 2011 and September 2012 were studied for immunohistochemistry analysis. HPIP expression was detected using real‐time reverse transcription PCR, Western blotting, and immunohistochemistry. Prognostic value of HPIP expression was examined using multivariate Cox regression analysis and Kaplan‐Meier method.

**Results:**

The result of Western blotting indicated that HPIP protein expression is significantly high in normal tissues compared to EC tissues (*P* < 0.001). The expression of HPIP was significantly associated with FIGO stage (*P* < 0.001), histological grade (*P* < 0.001), depth of myometrial invasion (*P* < 0.001), and lymph node metastasis (*P* = 0.033). Kaplan‐Meier analysis demonstrated that there was a significant difference in overall survival and disease‐free survival between the two groups of patients stratified by HPIP expression level (log‐rank, both *P* = 0.002). Patients with HPIP high expression had significantly shorter median survival time than those with HPIP low expression. Moreover, results of the multivariate analysis revealed that HPIP expression was an independent prognostic factor for predicting overall survival (*P* = 0.015) and disease‐free survival (*P* = 0.017) in patients with EC.

**Conclusion:**

The present study provides evidence that HPIP predicts EC progression and poor survival, highlighting its potential as a therapeutic target for EC.

## 1 INTRODUCTION

Endometrial cancer (EC) is one of the most common cancers in women worldwide.^1^ According to reports in the literature, the incidence of endometrial cancer in developed countries is higher than in developing countries. However, more and more women in China have been diagnosed with endometrial cancer in recent years.^2^, ^3^ Despite advances in the standard treatment for endometrial cancer, the prognosis in most patients with advanced endometrial cancer remains unsatisfactory. Therefore, it is imperative to further explore the occurrence and development of endometrial cancer progression and to identify therapeutic targets.

Hematopoietic pre‐B‐cell leukemia transcription factor‐interacting protein (HPIP) was originally identified by a yeast two‐hybrid screening of a hematopoietic cDNA library. According to the currently reported studies, HPIP exhibited an excessive expression status in numerous cancer tissues, while HPIP expression is involved in various aspects of cancer progression and predicts poor prognosis in cancer patients.^4^, ^5^, ^6^, ^7^, ^8^, ^9^, ^10^, ^11^, ^12^, ^13^, ^14^, ^15^ However, by reviewing the literature, we have not found a report on the expression of HPIP in endometrial cancer.

Therefore, our research aimed to explore the expression status of HPIP in endometrial cancer and its relationship with clinicopathological features. At the same time, further evidence was provided for the possibility of HPIP as a potential target for the treatment of endometrial cancer.

## 2 MATERIALS AND METHODS

### 2.1 Patient population

This study has been obtained authorization from the Ethical Committee of the Harbin Medical University Cancer Hospital. Patients of this study have signed written informed consent. In total, 113 endometrial cancer tissues were obtained from the Harbin Medical University Cancer Hospital. All patients received no treatment before surgery, and the cancer tissue samples were from patients who underwent gynecological surgery between August 2011 and September 2012. All patients underwent hysterectomy, bilateral salpingo‐oophorectomy, pelvic and/or para‐aortic lymphadenectomy, partial oophorectomy, and peritoneal lavage for cytology.

The follow‐up ranged from 4 to 74 months, with a median follow‐up of 62 months. All clinical information to be studied is summarized and listed in Table 1.

**Table 1:** Association analyses between the expression levels of HPIP and the clinicopathological characteristics of endometrial carcinoma

Variables	Patients	HPIP expression		*P* a
	n	Low	High	
All cases				
Age (y)				
<60	49	22	27	0.704
≥60	64	32	32	
Histological type				
Endometrioid	100	48	52	1.000
Nonendometrioid	13	6	7	
FIGO stage				
Ⅰ	77	52	25	<0.001
Ⅱ	15	1	14	
Ⅲ	17	1	16	
Ⅳ	4	0	4	
Histological grade				
G1	43	33	10	<0.001
G2	41	15	26	
G3	29	6	23	
Lymph node metastasis				
No	104	53	51	0.033
Yes	9	1	8	
Depth of myometrial invasion				
<50%	57	43	14	<0.001
≥50%	56	11	45	

Abbreviations: FIGO, International Federation of Gynecology and Obstetrics; G1, well differentiated; G2, moderately differentiated; G3, poorly differentiated; HPIP, hematopoietic pre‐B‐cell leukemia transcription factor‐interacting protein.

a Chi‐square test.

### 2.2 Western blot analysis

Nine samples were frozen in liquid nitrogen. The lysate was mixed proportionally with a protease inhibitor and used to cleave the extracted protein. The BCA method was then used to determine the protein concentration in the sample solution. The next step is electrophoresis separation, membrane transfer, and closure. After that, the primary antibody (anti‐HPIP, 1:300, Abcam, LLC) hybridization and the secondary antibody (anti‐β‐actin, Santa Cruz Biotechnology) hybridization were carried out, and the membrane was removed for exposure identification. This experiment is repeated three times.

### 2.3 Immunohistochemistry and evaluation

Detection of HPIP expression by immunohistochemistry. Paraffin specimens were sectioned for immunohistochemistry experiments. Then, dewax, rehydrate, and warm the bath. The antigen was repaired by heating with an autoclave and washed with PBS. The washed sections were incubated with an anti‐HPIP antibody (Abcam, catalogue number [ab176591]) at a dilution of 1:200 in a humid box and stored at 4°C overnight. The secondary antibody is then washed and added to detect bound antibodies. Sections were counterstained with hematoxylin, dehydrated, clarified, and sealed. The positive control for this experiment was a confirmed ovarian cancer wax block, while the negative control used rabbit serum instead of primary antibody staining.

The proportion of positive tumor cells was used as the criteria for staining score: 0 (positive tumor cells accounted for less than 5%); 1 (positive tumor cells accounted for 5‐25%); 2 (positive tumor cells accounted for 25‐75% ); 3 (positive tumor cells accounted for more than 75%). Microscopic staining intensity grading: 0 (no), 1 (weak), 2 (moderate), and 3 (strong). The proportional score is multiplied by the score of the staining grade to obtain the final score. The final score is expressed as low expression (0‐2) and high expression (≥3).

All immunohistochemical stains were scored by a professional pathologist who did not know the source of the sample.

### 2.4 Real‐time PCR

The expression of HPIP mRNA was quantified by RT‐PCR. Nine samples were used for real‐time PCR, including five endometrial cancer samples and four normal tissue samples. Total RNA was isolated using a TRIzol reagent (Wanlei) and converted to cDNA using the SuperScript III Platinum Kit (Invitrogen). Primers for HPIP are as follows: Forward, 5′‐ TTCTGGATGGCAGGAAGAT‐3; Reverse, 5′‐TCAAGGAGTCAAAGGAGGC‐3. The primer sequence for β‐actin as a reference is as follows: Forward, 5′‐CGGGAAATCGTGCGTGAC‐3; Reverse, 5′‐GTCAGGCAGCTCGTAGCTCTT‐3. Real‐time PCR was performed with SYBR® Fast qPCR Mix (TaKaRa). Relative HPIP abundance was determined by the $2^{-\Delta C_{\text{T}}}$ method. This experiment was repeated three times.

### 2.5 Statistical analysis

Analysis of HPIP expression in different clinicopathological differences by chi‐square test or Fisher's exact test. Survival analysis of the samples was verified by Kaplan‐Meier method and log‐rank test. The Cox model is used for multivariate analysis of independent factors associated with cancer. Data were expressed as mean ± SD for Western blot and RT‐PCR analysis. Data were expressed as mean ± SE for survival analysis. Statistical analysis was performed using SPSS 21.0 software (SPSS).

## 3 RESULTS

### 3.1 HPIP expression in patients with endometrial cancer

**Figure 1:**
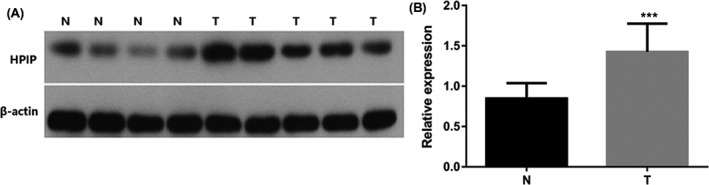
HPIP protein expression in normal and endometrial cancer tissues. A, Protein samples obtained from frozen normal endometrial tissues (N) and endometrial cancer tissues (T) were analyzed by Western blot analysis. Levels of β‐actin were used as an internal control; B, histogram of pooled data from N (n = 4) and ECs (n = 5). HPIP expression was elevated in ECs compared with N. The data are presented as mean ± SD (***P < 0.001)

**Figure 2:**
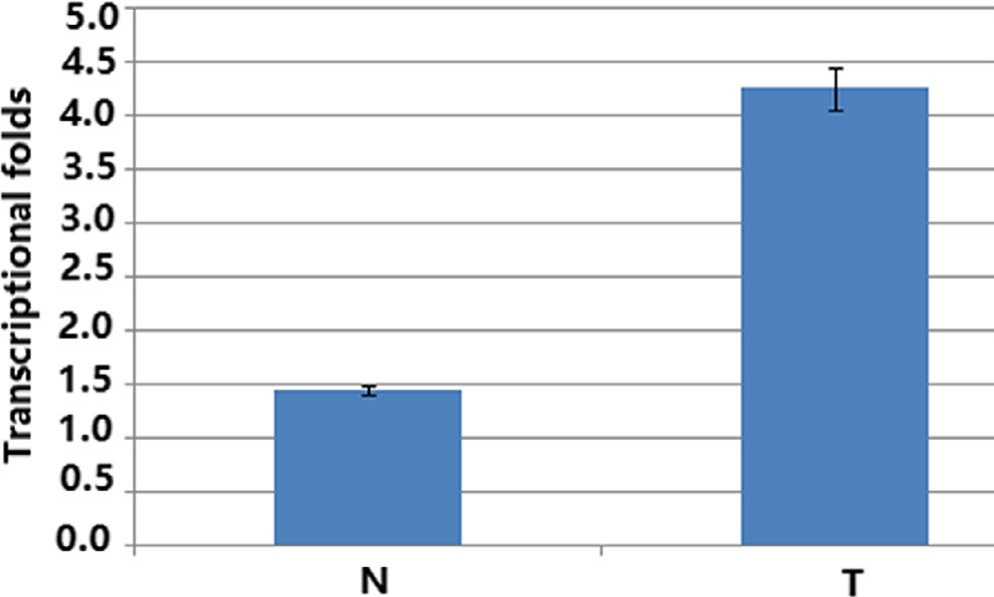
Histogram of HPIP mRNA expression in normal endometrial tissues (N) and endometrial cancer tissues (T). The levels of β‐actin were used as an internal control, and the HPIP mRNA expression was calculated by $2^{-\Delta \Delta C_{\text{t}}}$ method. HPIP mRNA expression was elevated in CCs compared with normal endometrial tissues. The data are presented as mean ± SD (*P* < 0.05)

A total of 113 endometrial cancers samples were studied in our research for immunohistochemistry analysis. The clinicopathological features of the patients participating in the study are summarized in Table 1. In addition, fresh trozen tissues from five samples of EC and four normal endometrial tissues were analyzed for HPIP expression using RT‐PCT and Western blot analysis. Western blotting results showed that the expression of HPIP was excessive at protein level in endometrial cancer samples (*P* < 0.001; Figure 1). Real‐time PCR results showed that the expression of HPIP at mRNA level in endometrial cancer tissues is much higher than that in normal endometrial tissues (*P* < 0.05, Figure 2).

### 3.2 HPIP expression is associated with clinicopathological feature in endometrial cancer

**Figure 3:**
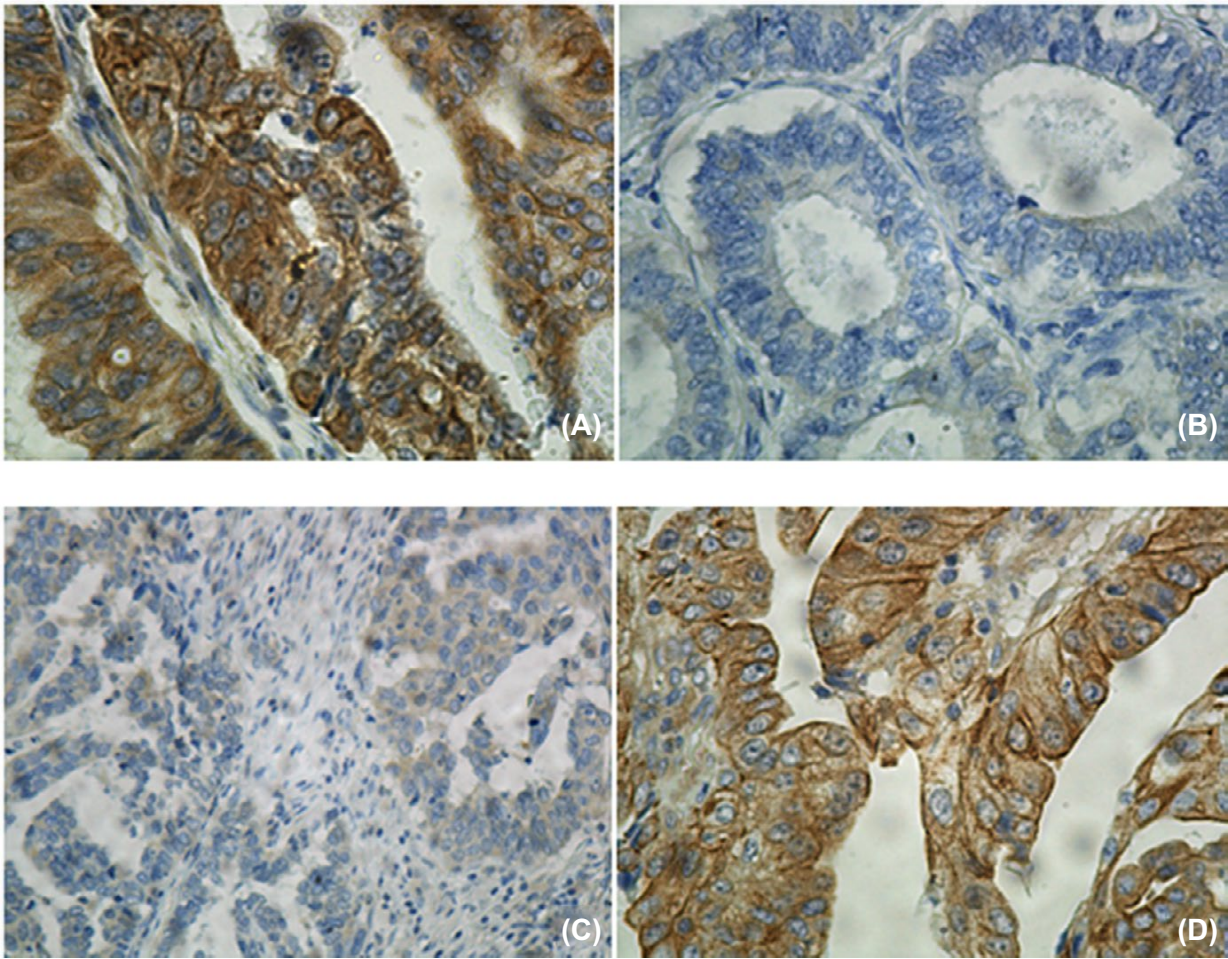
Immunohistochemical staining of HPIP in endometrial cancer specimens (EC). A, High expression of HPIP in a high‐grade endometrioid adenocarcinoma (×400); B, low expression of HPIP in a high‐grade endometrioid adenocarcinoma (×400); C, low expression of HPIP in a low‐grade serous endometrial carcinoma (×400); D, high expression of HPIP in a clear cell carcinoma of endometrium (×400)

To further examine the expression pattern of HPIP in patients with endometrial carcinoma, HPIP expression was detected using immunohistochemistry. Results of immunohistochemical staining are shown in Figure 3A‐D. As shown in Table 1, there are significant associations between HPIP expression and clinicopathological features of endometrial carcinoma. Patients could be classified into two patient subgroups: the patient group with the high expression level of HPIP and those with low expression level of HPIP. The two groups of patients did differ markedly in clinicopathological characteristics. Excessive expression of HPIP was clearly associated with high FIGO stage (*P* < 0.001), deep myometrial invasion (*P* < 0.001), high histological grade (*P* < 0.001), and lymph node metastasis (*P* = 0.033; Table 1).

### 3.3 Association between HPIP expression and prognosis of patients with endometrial cancer

**Figure 4:**
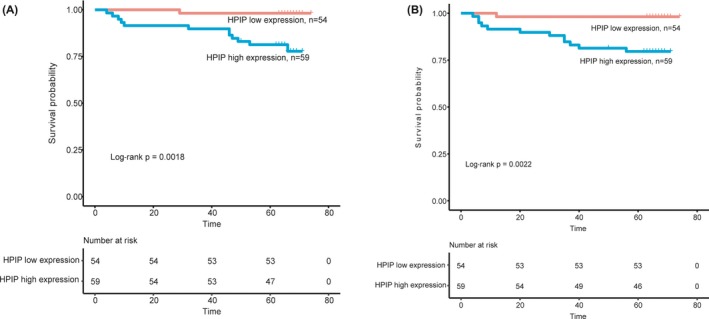
Association between HPIP expression and prognosis of patients with endometrial carcinoma. A, Kaplan‐Meier analysis of overall survival related to the expression of HPIP. Patients with high expression of HPIP had a poorer overall survival than those of patients with low expression of HPIP. B, Kaplan‐Meier analysis of disease‐free survival related to the expression of HPIP. Patients with high expression of HPIP had a poorer disease‐free survival than those of patients with low expression of HPIP

According to HPIP expression, patients could be classified into two patient subgroups: patient group with high expression level of HPIP (n = 59) and those with low expression level of HPIP (n = 54). Results of Kaplan‐Meier analysis suggested that there was significant difference in overall survival between the two groups of patients stratified by HPIP expression level (log‐rank *P* = 0.002; Figure 4A). HPIP‐overexpressed endometrial cancer patients had shorter median survival time (log‐rank *P* = 0.002; Table 2). We further evaluated the capability of the HPIP expression in predicting disease‐free survival. In consistent with the findings described above, the two groups of patients stratified by HPIP expression level showed significantly different disease‐free survival (log‐rank *P* = 0.002; Figure 4B). The disease‐free survival of the HPIP overexpression group was clearly shorter (log‐rank *P* = 0.002; Table 2).

**Table 2:** Univariate survival analysis of OS and DFS in 113 patients with endometrial carcinoma

Variables	n	OS			DFS		
		Mean ± SE (mo)	95% CI	*P* a	Mean ± SE (mo)	95% CI	*P* a
Age (y)							
<60	49	69 ± 2	65‐73	0.748	69 ± 2	64‐73	0.880
≥60	64	66 ± 2	63‐70		67 ± 1	64‐70	
Histological cell type							
Endometrioid	100	70 ± 1	67‐72	0.138	70 ± 1	67‐73	0.068
Nonendometrioid	13	62 ± 5	53‐72		63 ± 4	54‐71	
FIGO stage							
Ⅰ	77	72 ± 1	69‐74	0.003	71 ± 1	69‐74	0.002
Ⅱ	15	65 ± 4	57‐74		68 ± 2	64‐73	
Ⅲ	17	63 ± 4	54‐71		64 ± 4	56‐71	
Ⅳ	4	37 ± 15	8‐66		37 ± 15	8‐66	
Histological grade							
G1	43	72 ± 1	70‐74	0.459	73 ± 1	71‐75	0.291
G2	41	65 ± 2	60‐70		64 ± 3	58‐69	
G3	29	62 ± 3	55‐69				
Lymph node metastasis							
No	104	70 ± 1	68‐73	0.019	70 ± 1	68‐73	0.012
Yes	9	55 ± 8	38‐71		53 ± 9	35‐70	
Depth of myometrial invasion							
<50%	57	70 ± 1	68‐71	0.007	70 ± 1	69‐72	0.002
≥50%	56	65 ± 3	60‐70		65 ± 3	59‐70	
HPIP							
Low expression	54	73 ± 1	72‐75	0.002	73 ± 1	71‐75	0.002
High expression	59	63 ± 2	58‐68		61 ± 3	56‐67	

Abbreviations: FIGO, International Federation of Gynecology and Obstetrics; G1, well differentiated; G2, moderately differentiated; G3, poorly differentiated; HPIP, hematopoietic pre‐B‐cell leukemia transcription factor‐interacting protein; OS, overall survival; DFS, disease‐free survival.

a Log‐rank test.

### 3.4 Independence of HPIP expression from other clinicopathological factors

To assess whether prognosis prediction ability of HPIP expression is independent of other clinicopathological features of patients with endometrial carcinoma, we performed multivariate Cox regression analysis. As shown in Table 3, the results demonstrated that HPIP expression (HR = 12.614, CI = 1.636‐97.278, *P* = 0.015) was obviously related to overall survival, while HPIP expression (HR = 12.008, CI = 1.561‐92.375, *P* = 0.017) was markedly correlated with disease‐free survival of the endometrial carcinoma. Taken together, these results thus indicated that the predictive value of HPIP expression as a molecular biomarker is independent of other clinicopathological factors (Table 3).

**Table 3:** Multivariate survival analysis of OS and DFS in 113 patients with endometrial carcinoma

Variables	OS			DFS		
	Exp (B)	95% CI	*P* a	Exp (B)	95% CI	*P* a
FIGO stage	1.363	0.725‐2.564	0.337	1.364	0.728‐2.555	0.332
Lymph node metastasis	1.540	0.367‐6.460	0.555	1.501	0.359‐6.273	0.578
Depth of myometrial invasion	2.049	0.388‐10.816	0.542	2.300	0.450‐11.746	0.317
HPIP	12.614	1.636‐97.278	0.015	12.008	1.561‐92.375	0.017

Abbreviations: FIGO, International Federation of Gynecology and Obstetrics; G1, well differentiated; G2, moderately differentiated; G3, poorly differentiated; HPIP, hematopoietic pre‐B‐cell leukemia transcription factor‐interacting protein; OS, overall survival; DFS, disease‐free survival.

a Cox regression test.

## 4 DISCUSSION

In this study, we investigated whether HPIP is highly expressed in endometrial cancer tissues and its association with clinicopathological features. Furthermore, this study also explored the prognostic impact of HPIP expression on EC patients. Collectively, our experiments demonstrated that HPIP is indeed closely related to endometrial cancer and it could be used to index the prognosis and metastasis of endometrial cancer.

As shown in Figures 1 and 2, the consequence of real‐time PCR (mRNA levels) and Western blot (protein level) certified that excessive HPIP expression was found in EC samples nevertheless not found in normal samples. In this study, we performed data analysis on the results of immunohistochemistry of 113 endometrial cancer samples. Data analysis showed that overexpressed HPIP was highly correlated with clinicopathological features of endometrial cancer, including FIGO stage, myometrial invasion, histological grade, and lymph node metastasis. All of the above studies indicated that HPIP is involved in and plays a non‐negligible element in promoting cancer progression. We found that patients with HPIP high expression had worse disease‐free survival and overall survival through Kaplan‐Meier analysis and log‐rank analysis. As shown in Table 3, the results from the COX regression model data analysis directly demonstrated the close correlation between HPIP expression and patients overall survival (*P* = 0.015) and further confirmed that the excessive expression can promote disease‐free survival in a disappointing direction (*P* = 0.017). The above experimental results demonstrated that excessive expression of HPIP leads to a poor prognosis and can be used as a new molecular warning signal to assess cancer metastasis and prognosis in advance. As we have seen, our study is the first to explore the expression and impact of HPIP in endometrial cancer.

HPIP has been found to be overexpressed in a variety of cancers and participate in the development of tumors. In our previous research reports, HPIP overexpressed in ovarian cancer has been elucidated and the excessive expression of expression had a negative influence on the prognosis of the ovarian cancer. Later, our research on HPIP in cervical cancer also reached a consistent conclusion. In one paper, researchers used casein kinase 1α as a new key factor to identify HPIP. The researchers found that HPIP overexpression occurs in renal cell carcinoma; meanwhile, HPIP interacts with casein kinase 1α to participate in the development and metastasis of renal cell carcinoma. Bugide et al showed that overexpressed HPIP affects the prognosis of patients with primary breast cancer; Bugide et al studied HPIP in primary breast cancer and also found that the excessive expression caused a disproportionate prognosis and proposes to regulate HPIP‐mediated cancer cell migration by increasing the direct interaction of FAK phosphorylation of Y397 and activating FAK13. In vitro analysis revealed that HPIP prevents differentiation and proliferation of oral squamous cell carcinoma cells. A study from China uncovered that HPIP is expressed immoderately in liver cancer cells. Simultaneously, experiment to inhibit tumor growth by knocking out HPIP proved that HPIP can promote the growth of liver cancer cells by activating G2‐M15. Pan et al showed that HPIP is unduly expressed in non‐small‐cell lung cancer and accelerates cancer progression. Wang et al indicated that HPIP was up‐regulated in glioblastoma and identified a pivotal role for HPIP in the upgrowth of glioblastoma. HPIP was studied in colorectal cancer tissues and found that compared with normal tissue, the expression of HPIP in colorectal cancer tissues was pretty high, and HPIP accelerates evolvement of colorectal cancer cells and inhibited apoptosis. Regarding the study of HPIP in endometrial cancer tissues, our findings are in accordance with findings from various cancer tissues above. All of these findings exposed that HPIP takes part in tumorigenesis and progression. It further implied that HPIP can be a valuable biomarker for predicting cancer progression and prognosis.

Our research has verified that the excessive expression of HPIP is clearly correlated with clinicopathological features and survival rates of endometrial cancer. Based on the mechanisms of action of HPIP in tumors, several studies have explored the potential use of HPIP as a cancer treatment. For example, the above research on renal small cells, the results of HPIP and CK1α interaction in nude mice, concluded that HPIP may be used as a promising approach to the treatment of renal small‐cell carcinoma. Pan et al showed that HPIP knockdown in non‐small‐cell lung cancer cells can significantly inhibit tumor growth, tumor migration, and invasion by inhibiting Sonic Hedgehog signaling pathway and suggest the value of identifying it as a promising treatment for non‐small‐cell lung cancer. Zhang et al revealed that HPIP silencing has a positive effect on inhibiting TGF‐β1‐induced epithelial‐mesenchymal transition in human ovarian cancer cells.

In brief, our results indicated that HPIP is excessively expressed in endometrial cancer and that excessive expression of HPIP may be closely related to cancer progression and prognosis. This study provided strong support and evidence for HPIP as a new biomarker and the potential therapeutic target for endometrial cancer. But these findings require more extensive experiments to verify.

## AUTHORS' CONTRIBUTIONS

HF and FLM conceived and designed the experiments; LC, TTZ, SGL, and YW performed the experiments and analyzed the data; and HF and FLM wrote the paper. All authors read and approved the final manuscript.

## ETHICS APPROVAL AND CONSENT TO PARTICIPATE

This research was completed in compliance with the Helsinki Declaration. The data collection and analysis were carried out without disclosing patients’ identities.

## References

[1] Siegel RL , Miller KD , Jemal A . Cancer statistics, 2017. CA Cancer J Clin. 2017;67(1):7‐30. 28055103 10.3322/caac.21387

[2] Patch A‐M , Christie EL , Etemadmoghadam D , et al. Whole‐genome characterization of chemoresistant ovarian cancer. Nature. 2015;521(7553):489‐494. 26017449 10.1038/nature14410

[3] Zhou M , Zhang Z , Zhao H , Bao S , Sun J . A novel lncRNA‐focus expression signature for survival prediction in endometrial carcinoma. BMC Cancer. 2018;18(1):39. 29304762 10.1186/s12885-017-3983-0 PMC5756389

[4] Mao Y , Xu R , Liu X , Shi W , Han Y . Elevated fibrous sheath interacting protein 1 levels are associated with poor prognosis in non‐small cell lung cancer patients. Oncotarget. 2017;8(7):12186‐12193. 28086239 10.18632/oncotarget.14575 PMC5355335

[5] Bugide S , Gonugunta VK , Penugurti V , Malisetty VL , Vadlamudi RK , Manavathi B . HPIP promotes epithelial‐mesenchymal transition and cisplatin resistance in ovarian cancer cells through PI3K/AKT pathway activation. Cell Oncol (Dordr). 2017;40(2):133‐144. 28039608 10.1007/s13402-016-0308-2 PMC13001559

[6] Cao S , Sun J , Lin S , et al. HPIP: a predictor of lymph node metastasis and poor survival in cervical cancer. Onco Targets Ther. 2017;10:4205‐4211. 28894377 10.2147/OTT.S141248 PMC5584897

[7] Chen B , Zhao J , Zhang S , Zhang Y , Huang Z . HPIP promotes gastric cancer cell proliferation through activation of cap‐dependent translation. Oncol Rep. 2016;36(6):3664‐3672. 27748944 10.3892/or.2016.5157

[8] Feng Y , Xu X , Zhang Y , et al. HPIP is upregulated in colorectal cancer and regulates colorectal cancer cell proliferation, apoptosis and invasion. Sci Rep. 2015;5:9429. 25800793 10.1038/srep09429 PMC4371107

[9] Mai H , Xu X , Mei G , et al. The interplay between HPIP and casein kinase 1alpha promotes renal cell carcinoma growth and metastasis via activation of mTOR pathway. Oncogenesis. 2016;5(10):e260. 27694835 10.1038/oncsis.2016.44 PMC5117846

[10] Pan J , Qin Y , Zhang M . HPIP promotes non‐small cell lung cancer cell proliferation, migration and invasion through regulation of the Sonic hedgehog signaling pathway. Biomed Pharmacother. 2016;77:176‐181. 26796282 10.1016/j.biopha.2015.12.012

[11] Shi S , Zhao J , Wang J , Mi D , Ma Z . HPIP silencing inhibits TGF‐beta1‐induced EMT in lung cancer cells. Int J Mol Med. 2017;39(2):479‐483. 28075456 10.3892/ijmm.2017.2851

[12] Wang SC , Chai DS , Chen CB , Wang ZY , Wang L . HPIP promotes thyroid cancer cell growth, migration and EMT through activating PI3K/AKT signaling pathway. Biomed Pharmacother. 2015;75:33‐39. 26463629 10.1016/j.biopha.2015.08.027

[13] Wang Y , Li M , Meng F , Lou G . HPIP expression predicts chemoresistance and poor clinical outcomes in patients with epithelial ovarian cancer. Hum Pathol. 2017;60:114‐120. 27818289 10.1016/j.humpath.2016.10.015

[14] Wang Y , Meng F , Liu Y , Chen X . Expression of HPIP in epithelial ovarian carcinoma: a clinicopathological study. Onco Targets Ther. 2017;10:95‐100. 28053543 10.2147/OTT.S114884 PMC5189975

[15] Zhang GY , Liu AH , Li GM , Wang JR . HPIP silencing prevents epithelial‐mesenchymal transition induced by TGF‐beta1 in human ovarian cancer cells. Oncol Res. 2016;24(1):33‐39. 27178820 10.3727/096504016X14575597858654 PMC7838700

